# The integrated stress response regulates BMP signalling through effects on translation

**DOI:** 10.1186/s12915-018-0503-x

**Published:** 2018-04-03

**Authors:** Elke Malzer, Caia S. Dominicus, Joseph E. Chambers, Jennifer A. Dickens, Souradip Mookerjee, Stefan J. Marciniak

**Affiliations:** 1 Cambridge Institute for Medical Research (CIMR), University of Cambridge, Wellcome Trust/MRC Building, Hills Road, Cambridge, CB2 0XY UK; 2 Department of Medicine, University of Cambridge, Addenbrooke’s Hospital, Hills Rd, Cambridge, CB2 0SP UK

**Keywords:** GCN2, PPP1R15, BMP, ATF4, Translation, 4E-BP

## Abstract

**Background:**

Developmental pathways must be responsive to the environment. Phosphorylation of eIF2α enables a family of stress-sensing kinases to trigger the integrated stress response (ISR), which has pro-survival and developmental consequences. Bone morphogenetic proteins (BMPs) regulate multiple developmental processes in organisms from insects to mammals.

**Results:**

Here we show in *Drosophila* that GCN2 antagonises BMP signalling through direct effects on translation and indirectly via the transcription factor *crc* (dATF4). Expression of a constitutively active GCN2 or loss of the eIF2α phosphatase dPPP1R15 impairs developmental BMP signalling in flies. In cells, inhibition of translation by GCN2 blocks downstream BMP signalling. Moreover, loss of d4E-BP, a target of *crc*, augments BMP signalling *in vitro* and rescues tissue development *in vivo.*

**Conclusion:**

These results identify a novel mechanism by which the ISR modulates BMP signalling during development.

**Electronic supplementary material:**

The online version of this article (10.1186/s12915-018-0503-x) contains supplementary material, which is available to authorized users.

## Background

GCN2 belongs to a family of stress-sensing kinases that phosphorylate the alpha subunit of eukaryotic translation initiation factor 2 (eIF2α) to activate the integrated stress response (ISR) [1]. When eIF2α is phosphorylated, the translation of most messenger RNAs (mRNAs) is reduced to limit amino acid consumption; however, a small subset is translated more efficiently, including the mRNA encoding the transcription factor ATF4 [2, 3]. Targets of ATF4 aid survival by promoting amino acid import and the biosynthesis of aminoacyl-transfer RNAs (tRNAs) [1]. One ISR target gene encodes an eIF2α phosphatase called PPP1R15A (also called GADD34), which dephosphorylates eIF2α to restore protein synthesis and permit the translation of ISR targets [4–6].

The importance of the ISR during stress is well appreciated, but it also plays a less well-understood role during development. In mice, a lack of the ISR owing to mutation of eIF2α (eIF2α^S51A^) causes growth retardation *in utero* and perinatal death [7], while exaggeration of the ISR by deleting both eIF2α phosphatases (PPP1R15A and B) causes very early embryonic death [8]. Mutation of the ISR kinase PERK in humans and mice has multiple effects on development including skeletal dysplasia [9]. At least some of the developmental effects of the ISR are mediated by ATF4. Consequently, *Atf4*^−/−^ mice have impaired osteoblast differentiation and bone mineralisation [10]. We previously showed that ATF4 regulates protein secretion via the transcription factor CHOP [5] and that *Chop*^−/−^ mice have retarded bone formation [11]. The role of the ISR in osteogenesis may involve bidirectional crosstalk between eIF2α phosphorylation and bone morphogenetic protein (BMP) signalling. For example, treatment of primary bone cultures with BMP2 triggers endoplasmic reticulum stress and induces ATF4 in a PERK-dependent manner [12], while CHOP promotes differentiation of osteoblasts upon treatment with BMP [13].

How BMP and GCN2 signalling might interact is not known. Here, we use *Drosophila melanogaster* to identify a novel mechanism by which GCN2 regulates BMP-dependent MAD phosphorylation.

## Results

### Depletion of dPPP1R15 or dGCN2 alters wing venation

To understand the role of the ISR in tissue development, we used the model organism *Drosophila melanogaster*. It shares ISR components with mammals [14, 15], but its smaller genome reduces redundancy. We previously reported that changes in the expression of the eIF2α kinase dGCN2 or the eIF2α phosphatase dPPP1R15 impair fly development [15]. To determine which tissues are sensitive to altered ISR signalling, we have now expressed *ppp1r15* RNA interference (RNAi) under the control of a panel of tissue-selective drivers (Additional file 1: Figure S1A). Ubiquitous knockdown of *ppp1r15* or knockdown limited to the ectoderm markedly impaired larval development. In contrast, *ppp1r15* depletion in multiple tissues including the fat body, somatic muscle, salivary gland, midgut visceral mesoderm, eye, central nervous system (CNS), ring gland or heart had no detectable consequence for development. However, the use of the *escargot* driver *(esgGAL4)*, which is expressed in several tissues including the imaginal discs, caused larval delay at the third instar stage (Additional file 1: Figure S1B–D). Larvae expressing *esgGAL4*-driven *ppp1r15* RNAi (*esg > ppp1r15* RNAi) were followed until 21 days after egg laying (AEL) with less than 10% reaching adulthood. Similarly, using an *engrailed* driver (*enGAL4)* to express *ppp1r15* RNAi primarily in the posterior compartments of the imaginal discs also led to developmental delay (Additional file 1: Figure S1E). Larvae expressing *enGAL4*-driven *ppp1r15* RNAi (*en* > *ppp1r15* RNAi) were delayed, but approximately 45% reached adulthood by 14 days. Delayed larvae appeared phenotypically normal, continuing to feed and grow in size.

Since loss of the phosphatase dPPP1R15 would be expected to cause hyperphosphorylation of its substrate eIF2α, we hypothesised that loss of the eIF2α kinases might rescue the effects of *ppp1r15* RNAi. Indeed, depletion of the eIF2α kinase *perk* by RNAi driven either by *esgGAL4* or *enGAL4* largely rescued *ppp1r15* RNAi-expressing animals to adulthood (Additional file 1: Figure S1D, E). Similarly, although depletion of *gcn2* by RNAi driven either by *esgGAL4* or *enGAL4* caused a modest developmental delay, with only ≈ 70% of animals reaching adulthood by 14 days, *esgGAL4 > gcn2* RNAi partially rescued the developmental delay caused by *ppp1r15* knockdown (Additional file 1: Figure S1C–E).

These results revealed that the development of *Drosophila* could be impaired by a genetic perturbation predicted to enhance phosphorylation of eIF2α. This sensitivity displayed a restricted tissue distribution that included the imaginal discs but excluded much of the animals’ tissue mass. Raising animals on a high protein diet rather than standard food had no measurable effect on the frequencies of wing phenotypes or on the number of animals eclosing (not shown). Low protein diets led to fewer adults, but the frequency of each phenotype was unaffected. These findings suggested that protein deprivation was unlikely to account for the observed role of the ISR in our model.

**Fig. 1:**
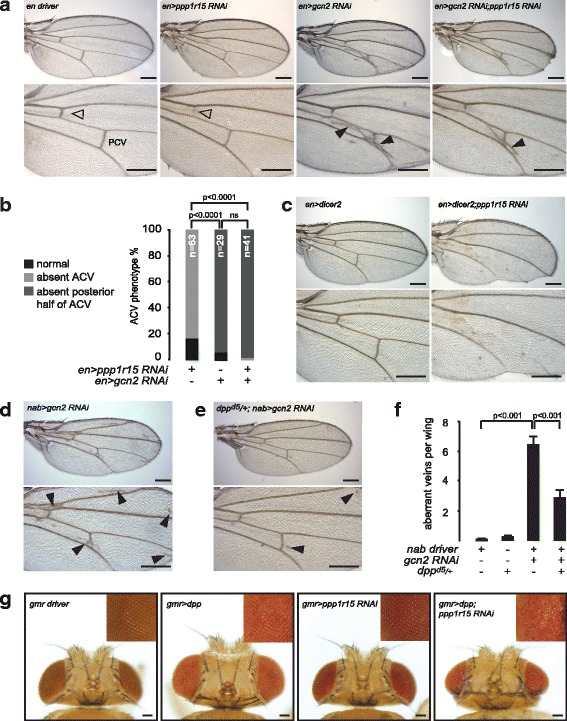
Depletion of dPPP1R15 or dGCN2 alters wing venation. **a** Representative photomicrographs (5× objective) of adult wings of the indicated genotypes. *Lower panels* are enlargements of the crossvein territories: anterior crossvein (ACV) (*open arrowhead*) and posterior crossvein (PCV). Note extra venation (*closed arrowheads*) in wings expressing *gcn2* RNAi. Scale bars = 250 μm. **b** Quantification of ACV phenotypes. For brevity, *enGAL4 > UAS-ppp1r15* RNAi is indicated as *en > ppp1r15* RNAi. *enGAL4 > UAS-gcn2* RNAi is indicated as *en > gcn2* RNAi. *n* denotes number of animals counted. *P* values calculated using *X*^2^ statistics with Bonferroni correction for multiple comparisons. **c** Representative photomicrographs of adult wings (5× objective) of the indicated genotypes. *en > dicer2* indicates *enGAL4 > UAS-dicer2*. *en > dicer2;ppp1r15* RNAi indicates *enGAL4 > UAS-dicer2;ppp1r15* RNAi. *Lower panels* are enlargements of the crossvein territories. Scale bars = 250 μm. **d**, **e** Representative photomicrographs of adult wings (5× objective) of the indicated genotypes. *nab > gcn2* RNAi indicates *enGAL4 > UAS-gcn2* RNAi. *dpp*^*d5/+*^*; nab > gcn2* RNAi indicates *Dpp*^*d5/+*^*; nab > UAS-gcn2* RNAi. *Lower panels* are enlargements of the crossvein territories. Note extra venation (*closed arrowheads*). **f** Quantification of wings from **d** and **e**. Scale bars = 250 μm. **g** Representative photomicrographs of adult eyes (dorsal view) of the indicated genotypes; *inset* shows zoom of eye. Scale bar = 200 μm

In most respects, *en > ppp1r15* RNAi animals appeared normal, although their wings lacked the anterior crossvein (ACV) (Fig. 1a, open triangle). By contrast, depletion of dGCN2 in the posterior compartment of the wing (*en > gcn2* RNAi) led to ectopic venation between longitudinal veins 4 (L4) and L5 (Fig. 1a, closed triangles). Frequently, *en > gcn2* RNAi animals lacked the posterior half of the ACV (Fig. 1a, b). When *en > ppp1r15* RNAi and *en > gcn2* RNAi were expressed together, the phenotype more closely resembled that of *en > gcn2* RNAi with ectopic venation between L4 and L5, and frequent absence of the posterior portion of the ACV (Fig. 1a, b). The effect of depleting dPPP1R15 on venation appeared to be dose-dependent, since augmentation of RNA interference by co-expression of dicer2 led to combined loss of the ACV, the posterior crossvein (PCV) and L4 (Fig. 1c).

When *gcn2* RNAi was driven by *nabGAL4*, ectopic venation was observed adjacent to the longitudinal veins (Fig. 1d, f, closed triangles). Because crossvein formation is sensitive to dpp (*Drosophila* BMP2/4) signalling [16], we examined the effect of manipulating dGCN2 and dPPP1R15 in animals with one hypomorphic allele of *dpp*, *dpp*^*d5*^ [17]. *dpp*^*d5/+*^ heterozygous animals retained normal wing venation (Additional file 1: Figure S1F), while *dpp*^*d5/+*^ animals showed significantly less ectopic venation caused by depleting dGCN2 with *nab > gcn2* RNAi (Fig. 1d–f). In contrast, loss of one wild-type allele (*dpp*^*d5/+*^*)* sensitised animals to depletion of *ppp1r15*, causing loss of posterior wing blade tissue and distal portions of L5 (Additional file 1: Figure S1F).

These results suggested that components of the ISR, specifically dGCN2 and dPPP1R15, could modulate wing imaginal disc development, and that this might involve effects on dpp/BMP signalling. In support of this, we also observed that depletion of dally, a cell-surface glypican involved in dpp signalling [18], also interacted genetically with dPPP1R15 and dGCN2. Alone, expression of *dally* RNAi using the *nab* driver had no effect on wing venation, but when combined with knockdown of *ppp1r15*, it exacerbated the loss of wing blade tissue and, once again, led to loss of distal portions of L5 (Additional file 1: Figure S1F). When combined with *nab* > *gcn2* RNAi, depletion of *dally* caused disorganised venation (not shown).

Overgrowth of the eye reports on elevated dpp signalling [19]. We therefore tested the effect of depleting dPPP1R15 in the eye using a *gmrGAL4* driver (Fig. 1g). As expected, overexpression of dpp in the eye led to eye overgrowth. Knockdown of dPPP1R15 alone had no detectable effect on eye development, but when combined with overexpression of dpp, it rescued eye growth to a normal size, albeit with a rough eye phenotype.

These observations suggested that the developmental effects of modulating the ISR were sensitive to the intensity of dpp signalling, revealing a novel genetic interaction between the ISR and BMP pathways during fly development.

### dPPP1R15 or dGCN2 affects MAD phosphorylation in the developing wing

**Fig. 2:**
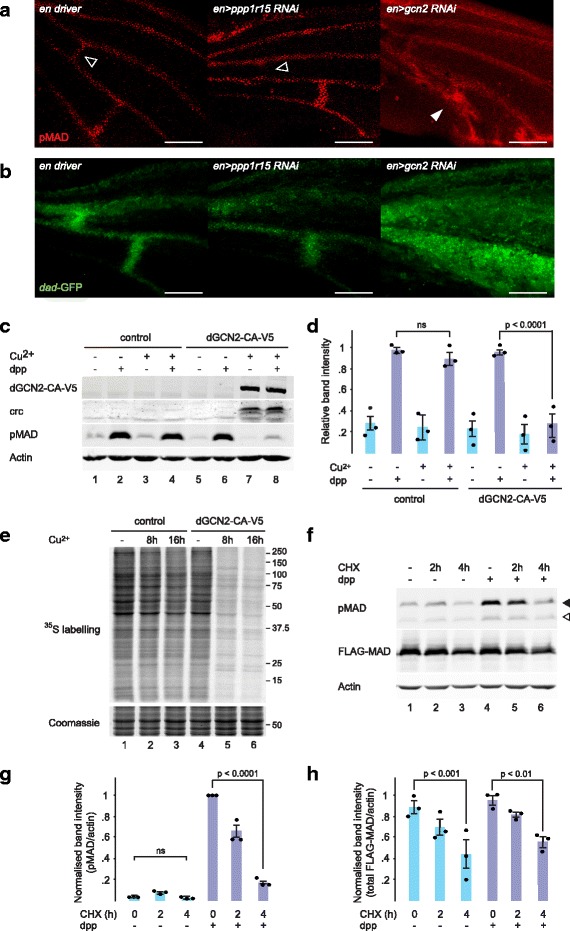
dPPP1R15 or dGCN2 affects MAD phosphorylation in the developing wing. **a** Representative fluorescence micrographs of pupal wings of the indicated genotypes at 30 h after pupariation *stained red* for pMAD. *Open arrowheads* indicate ACV territory. *Closed arrowheads* indicate ectopic pMAD signal. Scale bars = 100 μm. **b** Representative fluorescence micrograph of pupal wings of the indicated genotypes at 30 h after pupariation. *Green fluorescence* indicates activation of the *dad*-*GFP.N* reporter. Scale bars = 100 μm. **c** Immunoblot of cell lysates: lanes 1–4, S2 cells stably transfected with V5.pMT-Puro; lanes 5–8, S2 cells stably transfected with dGCN2-CA-V5.pMT-Puro. *Cu*^*2+*^ indicates treatment with 0.7 mM copper sulphate for 16 h; *dpp* indicates treatment with 1 nM Dpp for 1 h prior to lysis. dGCN2-CA-V5 was detected with anti-V5 antibody. crc, pMAD and actin were detected using specific antibodies. **d** Quantification of pMAD staining in **c** with strongest signal with each experiment set as 1. *n* = 3. *P* value calculated using analysis of variance (ANOVA) with Bonferroni *post hoc* testing. **e** S2 cell lysates: lanes 1–3, V5.pMT-Puro S2 cells; lanes 4–6, dGCN2-CA-V5.pMT-Puro S2 cells. *Cu*^*2+*^ indicates treatment with 0.7 mM copper sulphate for the indicated times. ^35^S-labelled cysteine and methionine were added to cells for 10 min prior to lysis. ^*35*^*S-labelling* indicates autoradiograph. Coomassie staining served as a loading control. **f** Immunoblot of S2 cell lysates expressing FLAG-MAD. *CHX* indicates treatment with 14 μg/ml cycloheximide for the indicated times. *dpp* indicates treatment with 0.5 nM dpp for 1 h prior to lysis. FLAG-MAD was detected with an anti-FLAG antibody. pMAD and actin were detected with specific antibodies. *Filled arrowhead* indicates phosphorylated MAD-FLAG; *open arrowhead* indicates endogenous pMAD. **g** Quantification of phosphorylated FLAG-MAD (pMAD) and (**h**) total FLAG-MAD from **f**, both normalised to actin signal with the strongest signal in each experiment set as 1. *n* = 3. *P* value calculated using ANOVA with Bonferroni *post hoc* testing

To define the effects of the ISR on more proximal readouts of dpp signalling, we next examined MAD phosphorylation in pupal wings. During pupation, longitudinal veins are specified by epidermal growth factor receptor and dpp signalling [20]. After the longitudinal veins have formed, the ACV and PCV are generated in response to Dpp that is transported from the adjacent longitudinal veins [21, 22]. As expected, 30 h after pupariation, pMAD staining was detected in the presumptive ACV and PCV territories of driver control wings (Fig. 2a, left panel). When dPPP1R15 was knocked down in the posterior compartment of the wing using *en > ppp1r15* RNAi, pMAD staining was evident in the PCV provein but was absent from ACV territory (Fig. 2a, middle panel, ACV territory indicated by open triangle), whereas when dGCN2 was instead depleted in the posterior compartment using *en > gcn2* RNAi, ectopic pMAD staining was detected between the L4 and L5 proveins (Fig. 2a, right panel, closed triangle). These changes in the distribution of MAD phosphorylation correlated well with the venation phenotypes observed in the adult wings of escapers (Fig. 1a).

To determine if the altered distribution of pMAD had functional consequences, we used a reporter comprising the promoter of a dpp-sensitive gene, *dad*, fused to the coding sequence of green fluorescent protein (*GFP)* [23]. As expected, in driver controls the GFP reporter signal was detected at the regions of the ACV and PCV proveins 30 h after pupariation (Fig. 2b, left panel). When *ppp1r15* was knocked down by *en > ppp1r15* RNAi, the GFP signal was undetectable in the ACV provein territory (Fig. 2b, middle panel), but when dGCN2 was depleted with *en > gcn2* RNAi, widespread ectopic reporter activation was seen, especially in the L4–L5 intervein region, and there was broadening of the GFP signal into the L3–L4 intervein region (Fig. 2b, right panel). Together, these data show that the precise arrangement of dpp signalling required for normal vein distribution in the pupal wing is dependent upon an intact ISR.

Because our *in vivo* studies had suggested that MAD phosphorylation is inhibited by activation of the ISR, we next turned to an *in vitro* model of dpp signalling to determine the mechanism of this interaction. Schneider 2 (S2) cells were generated to conditionally express a constitutively active dGCN2 tagged with the V5 epitope, dGCN2-CA-V5. In the absence of dGCN2-CA-V5, treatment with dpp caused robust phosphorylation of MAD (Fig. 2c, d). Induction of dGCN2-CA-V5 for 16 h was sufficient to activate the ISR as evidenced by expression of the transcription factor crc (dATF4). Remarkably, expression of dGCN2-CA-V5 abolished dpp-induced phosphorylation of MAD (Fig. 2c, lanes 7 and 8; Fig. 2d).

Activation of the ISR inhibits translation initiation [24]. Metabolic labelling with ^35^S-methionine and cysteine confirmed that expression of dGCN2-CA-V5 for 8 or 16 h reduced global translation (Fig. 2e). It seemed plausible that loss of total MAD protein might therefore contribute to the loss of pMAD following induction of dGCN2-CA-V5. There are no antibodies that detect total MAD, so to estimate its half-life we transfected S2 cells with FLAG-tagged MAD and inhibited protein synthesis with cycloheximide (Fig. 2f–h). Consistently, the level of total FLAG-MAD had halved by 4 h after inhibiting translation but was insensitive to dpp (Fig. 2f, h). The levels of pMAD and phosphorylated FLAG-MAD were much lower than half of their starting level by 4 h after treatment with cycloheximide (Fig. 2f, g). These results indicate that activation of dGCN2 is sufficient to inhibit global protein synthesis and that inhibition of translation is sufficient to decrease the levels of both MAD and pMAD. The apparently preferential effect of translational attenuation on the levels of pMAD suggested, however, that additional short-lived proteins may be required for efficient MAD phosphorylation or that pMAD is preferentially destabilised.

### crc regulates wing venation and antagonises MAD phosphorylation

crc is a bZIP transcription factor sharing sequence and functional homology with mammalian ATF4 [25, 26]. In order to confirm activation of the ISR, we generated an antibody able to detect endogenous crc by western blot (Fig. 2c and Additional file 2: Figure S2). This technique recognised a doublet of 65–70 kDa. After *in vitro* treatment with lambda phosphatase, crc doublets collapsed to a single band, indicating that, like ATF4, crc is a phosphoprotein (Additional file 2: Figure S2). Similar to ATF4, the 5′ untranslated region (5’UTR) of the *crc* mRNA contains several small upstream open reading frames (uORFs), the last of which overlaps out of frame with the crc coding sequence (Additional file 2: Figure S2B). To confirm the observation by Kang et al. (2015) [26] that translation of *crc* is regulated in a manner similar to that of ATF4, we generated a reporter construct comprising the 5’UTR of *crc* fused to the coding sequence of luciferase. The reporter or a control consisting of a luciferase coding sequence lacking the *crc* 5’UTR was expressed in mammalian human embryonic kidney 293T (HEK293T) cells, and the ISR was activated using tunicamycin (Additional file 2: Figure S2C). The translation of the *crc*-reporter luciferase mRNA rose upon treatment with tunicamycin, while translation of the control fell. The ISR mediates its inhibitory effects on global translation through phosphorylation of eIF2α, rendering it an inhibitor of its own guanine nucleotide exchange factor, eIF2B [27]. The inhibition of eIF2B is also ultimately responsible for the increased translation of ATF4. These effects can be overcome in mammalian cells by the eIF2B-activating drug ISRIB [28, 29]. We therefore treated the HEK293T cells with ISRIB and observed a selective reduction of the translation of the *crc*-luciferase reporter (Additional file 2: Figure S2C).

We had previously shown that overexpression of the ISR kinase dPERK in the eye imaginal disc *(gmr > perk)* impairs eye development [14]. To test if this effect of the ISR on development might be mediated by crc, we expressed *gmr > crc* RNAi simultaneously with *gmr > perk* (Additional file 2: Figure S2D). This rescued eye growth and confirmed crc as a mediator of the ISR in *Drosophila*.

**Fig. 3:**
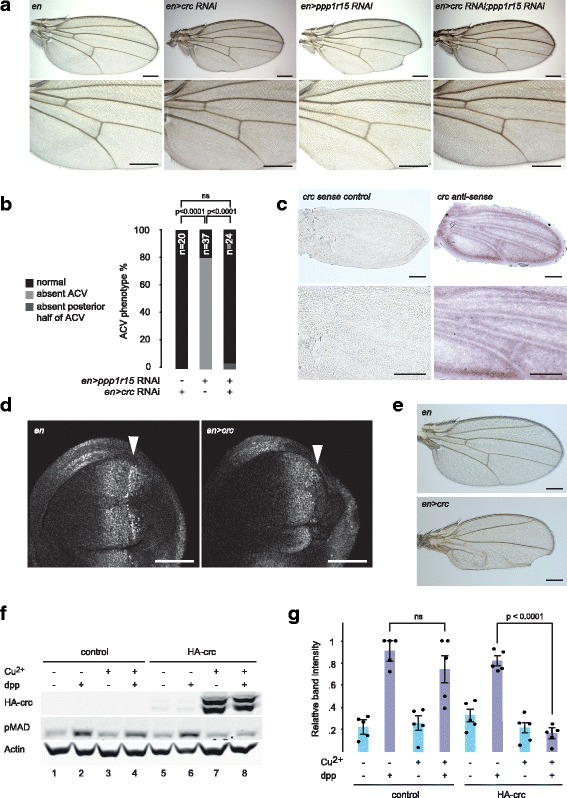
crc regulates wing venation and antagonises MAD phosphorylation. **a** Representative photomicrographs (5× objective) of adult wings of the indicated genotypes. *En* indicates *enGAL4* driver control. *en > crc* RNAi indicates *enGAL4 > UAS-crc* RNAi. *en > ppp1r15* RNAi indicates *enGAL4 > UAS-ppp1r15* RNAi. *en > ppp1r15* RNAi*;crc* RNAi indicates *enGAL4 > UAS-crc RNAi;UAS-ppp1r15* RNAi. *Lower panels* are enlargements of the crossvein territories. Scale bars = 250 μm. **b** Quantification of ACV phenotype in **a**. *P* values calculated using *X*^2^ statistics with Bonferroni correction for multiple comparisons. **c** *In situ* hybridisation of w^1118^ pupal wings with sense or antisense probes to residues 1405–1900 of *crc* transcript A. Scale bars = 250 μm. **d** Representative fluorescence micrograph (40× objective) of wing imaginal discs: signal = pMAD. *En* indicates *enGAL4* driver control. *en > crc* indicates *enGAL4 > UAS-HA-crcA*. *Orientation*: left = anterior. *Arrowhead* indicates expected position of posterior pMAD zone. Scale bars = 50 μm. **e** Representative photomicrographs of adult wings of the indicated genotypes. *En* indicates *enGAL4* driver control. *en > crc* indicates *enGAL4 > UAS-crc*. Scale bars = 250 μm. **f** Immunoblot of S2 cell lysates: lanes 1–4, S2 cells stably transfected with HA.pMT-Puro; lanes 5–8, S2 cells stably transfected with HA-crcA.pMT-Puro. *Cu*^*2+*^ indicates treatment with 0.7 mM copper sulphate for 24 h. *dpp* indicates treatment with 0.5 nM dpp for 1 h prior to lysis. HA-crc was detected with anti-HA antibody. pMAD and actin were detected using specific antibodies. **g** Quantification of pMAD staining in **f** with highest signal per experiment set as 1. *n* = 5. *P* value calculated using analysis of variance (ANOVA) with Bonferroni *post hoc* testing

The ACV was unaffected when crc was depleted in the developing wing using *en > crc* RNAi, but *en > crc* RNAi suppressed the ACV phenotype of *en > ppp1r15* RNAi (Fig. 3a, b). In the presence of dicer2, RNAi against *crc* driven by *enGAL4* caused loss of the ACV’s posterior portion similar to that observed with depletion of *gcn2* (Additional file 2: Figure S2E, F). Similar results were obtained with a whole-wing *nab* driver (Additional file 2: Figure S2G). *In situ* hybridisation was performed to examine the distribution of *crc* mRNA in the developing wing (Fig. 3c and Additional file 2: Figure S2H). In wing imaginal discs, *crc* expression was widespread throughout the pouch (Additional file 2: Figure S2H), while the pupal wing showed staining along the wing margin and surrounding the presumptive longitudinal and crossveins (Fig. 3c). Similar results were obtained using a second probe targeting a separate region of the *crc* mRNA (not shown).

Next, we generated transgenic flies overexpressing crc. Wing imaginal discs expressing crc in the posterior compartment using the *enGAL4* driver showed reduced tissue mass and an absence of pMAD in the posterior portion of the disc (Fig. 3d). In adult wings, crc expression in the posterior compartment of the wing reduced blade size and impaired venation (Fig. 3e). When expressed in the whole wing using *nabGAL4*, crc generated smaller wings with evidence of inadequate crossvein L3, L4 and L5 formation (Additional file 2: Figure S2I). These results indicated that crc can modify signals regulating venation *in vivo*. To examine this further, we generated S2 cells that conditionally expressed crc. As we had seen for dGCN2, crc expression blocked the phosphorylation of MAD caused by dpp (Fig. 3f, g).

These results suggest that crc mediates at least some of the inhibition of BMP signalling that is caused by eIF2α hyperphosphorylation, and that crc is capable of attenuating MAD phosphorylation.

### 4E-BP mediates part of the crc effect on wing venation and MAD phosphorylation

**Fig. 4:**
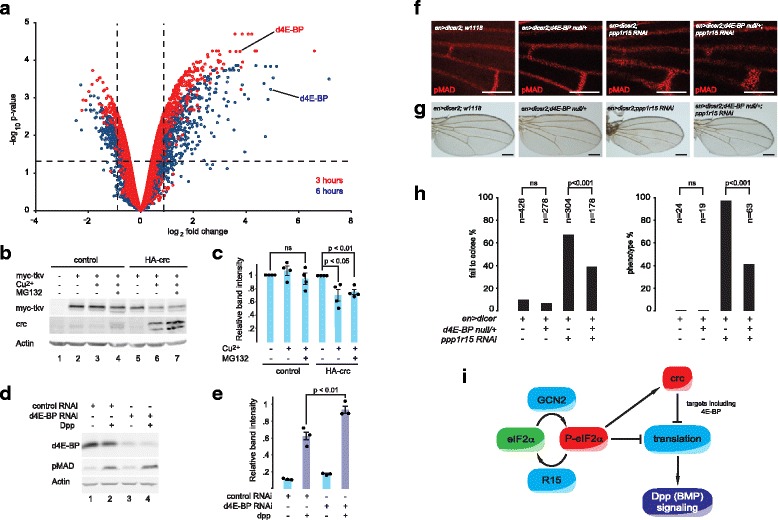
4E-BP contributes to the inhibition of MAD phosphorylation. **a** Microarray analysis of transcriptional changes caused by expression of crc in S2 cells. Volcano plot of transcriptional profiles of HA-crcA.pMT-Puro S2 stable cells relative to HA.pMT-Puro S2 stable cells, each treated with 0.7 mM copper sulphate for 3 h (*red symbols*) or 6 h (*blue symbols*). *Vertical broken lines* indicate 2^−/+ 0.7^-fold change. *Horizontal broken line* indicates *P* = 0.05 threshold. d4E-BP is indicated at 3 h (*red*) and 6 h (*blue*). **b** Immunoblot of cell lysates expressing myc-Tkv in the absence or presence of crc. **c** Quantification of **b**, samples normalised to no Cu^2+^ for each cell line. *n* = 3. *P* value calculated using ANOVA with Bonferroni *post hoc* testing. **d** Immunoblot of S2 cell lysates to assess the effect of d4E-BP small interfering RNA (RNAi) on MAD phosphorylation caused by 0.5 nM Dpp concentrations. **e** Quantification of **d**. *n* = 3. *P* value calculated using ANOVA with Bonferroni *post hoc* testing. **f** Representative fluorescence micrographs of pupal wings of the indicated genotypes at 30 h after pupariation *stained red* for pMAD. Scale bars = 100 μm. **g** Representative photomicrographs (5× objective) of adult wings of the indicated genotypes. Scale bars = 200 μm. **h** Quantification of animals from **g**. *Left graph* indicates proportion of animals failing to eclose by 14. *Right graph* indicates frequency of wing vein phenotype if eclosing adults. *P* values calculated using *X*^2^ statistics with Bonferroni correction for multiple comparisons. **i** Schematic of interaction between integrated stress response (ISR) and BMP signalling. eIF2α is phosphorylated by GCN2 to P-eIF2α; PPP1R15 (*R15*) dephosphorylates P-eIF2α. P-eIF2α directly inhibits most cap-dependent translation of mRNAs, but induces expression of *crc* (*Drosophila* ATF4). Targets of crc further affect translation, e.g. 4E-BP antagonises translation of some mRNAs. Ongoing translation is necessary for efficient BMP signalling, and so repression of protein synthesis by the ISR inhibits BMP signalling

To characterise the genes whose expression was altered by crc, we performed transcriptional profiling of S2 cells expressing crc for 3 or 6 h (Fig. 4a). As expected, pathway analysis showed crc to induce genes involved in amino acid sufficiency and ribosome function (Additional file 3: Figure S3 and Additional file 4: Tables S1, S2). Gene Ontology (GO) term enrichment revealed the induction of many additional factors affecting translation (Additional file 4: Tables S1, S2). Transcripts that were significantly reduced included positive regulators of the cell cycle and nucleic acid biogenesis. Similar transcriptional changes were induced by expression of dGCN2-CA-V5 (Additional file 3: Figure S3 and Additional file 4: Tables S9, S10). In contrast to dGCN2-CA-V5, an inactive mutant of dGCN2 (dGCN2-K552R-V5) failed to induce genes involved in ribosome biogenesis, suggesting that increased protein synthetic load was not responsible for these effects (not shown).

The preponderance of regulators of translation among the crc-sensitive transcripts raised the possibility that MAD phosphorylation might be affected in crc-expressing cells through additional changes to protein synthesis over and above those caused by eIF2α phosphorylation. An antibody capable of detecting the endogenous type I BMP receptor Tkv is lacking, so to determine if crc-induced inhibition of translation might affect Tkv protein levels, we expressed myc-tagged Tkv in the inducible crc-expressing S2 cells. Crc significantly suppressed myc-Tkv protein levels by approximately 20%, and this could not be rescued by inhibition of the proteasome with MG132, indicating an effect on synthesis rather than proteasomal degradation of the protein (Fig. 4b, c).

Although it is likely that many crc-sensitive factors cooperate to achieve this effect on protein synthesis, we chose to focus on eIF4E-binding protein (4E-BP), as it was one of the most highly induced negative regulators of translation in our transcriptional profiling (Fig. 4a and Additional file 4: Tables S1, S2, S9, S10). The *Drosophila* homologue of 4E-BP (Thor) was up-regulated 30-fold at the mRNA level after 6 h of crc expression (Fig. 4a and Additional file 3: Figure S3D). This induction was confirmed at the protein level by western blot (Additional file 3: Figure S3E). Depletion of d4E-BP by RNAi in S2 cells significantly augmented dpp-induced MAD phosphorylation, suggesting that d4E-BP exerts a tonic inhibition on dpp-MAD signalling (Fig. 4d, e).

To test the relevance of this effect *in vivo*, we generated animals haploinsufficient for *d4E-BP*. In *d4E-PB*^*null*/+^ [30], the phosphorylation of MAD within the pupal wing vein territories was normal, as were the adult wing veins (Fig. 4f and g). However, loss of one *d4E-BP* allele significantly rescued both the numbers of animals eclosing and the normal formation of the ACV in wings depleted of *ppp1r15* in the posterior compartment using *en > ppp1r15* RNAi (Fig. 4f–h). These findings indicate that the impaired dpp-MAD signalling observed in this model is sensitive to the levels of d4E-BP. Taken together, our observations suggest that targets of crc that regulate translation contribute to the inhibition of dpp signalling during development.

## Discussion

We have shown that the ISR modulates tissue morphogenesis through the regulation of dpp-induced MAD phosphorylation. In wing tissue, this mechanism is driven primarily by the eIF2α kinase dGCN2. These repressive effects are achieved directly by the reduction in translation that accompanies phosphorylation of eIF2α, and indirectly by the induction of the transcription factor crc (dATF4) and its targets including d4E-BP (Fig. 4i). Since the ISR is conserved between metazoans, our findings may have wider significance in developmental biology.

Developmental signals orchestrate tissue patterning by following predetermined programmes. Environmental factors also have an impact on development, and so crosstalk between stress signalling and developmental pathways is necessary. It is known that overexpression of non-phosphorylatable mutants of eIF2α accelerates development of enlarged adult female flies, while expression of a phosphomimetic eIF2α delays larval development [31]. We previously reported that depletion of the eIF2α phosphatase dPPP1R15 causes a developmental delay similar to that of phosphomimetic eIF2α [14]. We have now shown that expression of dPPP1R15 is necessary for larval development only in specific larval tissues, including the imaginal discs, and shares an antagonistic relationship with dGCN2.

*In vitro* studies indicate that inhibition of protein synthesis mediates some of the inhibitory effects of dGCN2 on BMP signalling, reflecting the short half-lives of components of the BMP signalling cascade. Vein formation in the fly wing is governed by BMP signalling. Dpp (the *Drosophila* BMP2/4 homologue) binds to the type I receptors, Tkv or Sax, and type II receptor Punt to phosphorylate and activate the transcription factor MAD [32]. Crossvein morphogenesis requires secretion of dpp from nearby longitudinal veins and its chaperoning by the molecules tsg, cv and sog, which are subsequently degraded by Tlr to release dpp at sites defined by high levels of cv-2 [21, 33]. The formation of the dpp gradient also requires the expression of extracellular glypicans, such as dally, and their post-translational modification by enzymes including sulfateless [34, 35]. Changes in the expression levels of at least some of these components may contribute to impaired BMP signalling during activation of the ISR. *dally* RNAi had a more dramatic effect on wing development when expressed with *ppp1r15* RNAi, compared with *ppp1r15* RNAi in flies with one hypomorphic allele of *dpp*^*d5*^. This might relate to differences in the degree to which *dally* and *dpp* were depleted, but it might also reflect the dual role of dally in both stabilising and dispersing dpp in the extracellular space and as a co-receptor involved directly in dpp signalling [36]. Examination of wing imaginal discs has not yet revealed dramatic effects of the ISR on signalling via the wnt or hedgehog pathways (not shown), but further studies are necessary before the regulation of developmental signalling by the ISR can be said to show specificity towards the BMP pathway.

crc, the *Drosophila* homologue of ATF4, also inhibits MAD phosphorylation. The large number of genes sensitive to crc suggests that its effect on BMP signalling may be multifaceted. Our data reveal, however, that part of this effect is mediated by the induction of d4E-BP. Of note, ATF4 binding sites have recently been identified within the *d4E-BP* gene [37]. By binding to eIF4E, 4E-BP prevents assembly of eIF4F and so selectively inhibits cap-dependent translation [38]. Interestingly, expression of a hyperactive mutant of d4E-BP in the wing has been shown to result in selective loss of the ACV, although the mechanism was unknown [39]. How elevated d4E-BP levels inhibit phosphorylation of MAD in the absence of detectable effects on global translation rates is unclear. It is plausible that the extent of translational attenuation may vary among cap-dependent mRNAs, and, in such a model, as levels of available eIF4E decline, some mRNAs might compete more efficiently than others for a limited supply of the eIF4F. Such sensitivity could explain some of the effects we have described, although the mRNAs responsible for altered MAD phosphorylation have yet to be fully identified. Nevertheless, there are numerous instances in which d4E-BP selectively regulates mRNA translation. For example, insulin signalling inhibits neurotransmitter release via d4E-BP-mediated repression of *complexin* mRNA translation [40], while dietary restriction enhances the expression of mitochondrial respiratory components by inducing d4E-BP [41]. Indeed, there is emerging evidence in *Drosophila* that ISR-induced d4E-BP plays a role in biasing translation during infection [42], development and aging [37].

Mice generated to be insensitive to the ISR kinases owing to mutation of the target serine 51 of eIF2α revealed a role for the ISR in mammalian development [7]. Homozygous pups were growth retarded and died from hypoglycemia due to impaired gluconeogenesis, while heterozygous animals developed diabetes if fed high-fat chow owing to impaired pancreatic ß-cell survival.

Pulmonary arterial hypertension (PAH) is a family of diseases that predominantly affects young adults and carries a high mortality. Although most cases are idiopathic, in 70% of familial cases and 20% of sporadic cases heterozygous germline mutations are identified in the type II BMP receptor (*BMPR2*) [43–45]. The penetrance of the *BMPR2* mutation is highly variable, suggesting that additional modifying factors must exist. Recently, two rare subtypes of PAH, pulmonary veno-occlusive disease (PVOD) and capillary haemangiomatosis, were shown to be caused by mutations of *EIF2AK4*, which encodes the kinase GCN2 [46, 47]. Interestingly, *BMPR2* mutations have also been associated with PVOD, suggesting that similar mechanisms may underlie typical PAH and PVOD [48, 49]. It is tempting to speculate that the mechanism linking GCN2 and BMP signalling that we have described here might have relevance to PAH. Why loss of GCN2-mediated inhibition of BMP signalling should cause a disorder more commonly associated with insufficient SMAD phosphorylation is intriguing. However, mammalian BMP signalling is more complex than that of insects, and it is known that loss of signalling via one BMP type II receptor in pulmonary artery smooth muscle cells can lead to excessive signalling through other type II receptors [50]. Further studies will be necessary to determine if the ISR regulates BMP signalling within the mammalian pulmonary vasculature.

## Conclusion

In summary, we report a novel mechanism for the modulation of BMP signalling by the ISR. This involves direct modulation of translation initiation through eIF2α phosphorylation and indirect effects via the crc-d4E-BP axis. This raises the possibility that pharmacological manipulation of the ISR may represent a therapeutic approach for the regulation of BMP signalling.

## Methods

### *Drosophila* genetics

The following strains were obtained from the Vienna Drosophila RNAi Center: *ppp1r15* (RNAi #1: 15238; RNAi #2: v107545); *gcn2* (v103976); *crc* (v109014); *dally* (14136) and 51D background as a control line. Stocks obtained from the Bloomington Drosophila Stock Center (National Institutes of Health (NIH) P40OD018537) were *UAS-dally* (5397); *engrailed-Gal4* (6356); *UAS-dicer2*; *en-Gal4, UAS-eGFP* (25752); *UAS-dpp* (1486); *dpp*^*d5*^ (2071); *dpp*^*hr56*^ (36528); *GMR-Gal4* (1104). Other lines were supplied as follows: isogenic w^1118^ line; w^1118^; *if/CyO*; *gmr-GAL4/TM6B* (from Dr S Imarisio, University of Cambridge); *escargot*^*NP7397*^*-Gal4; yw.hs-flp*^*122*^; *Act5c > y*^*+*^ *> Gal4, UAS-GFP*; *MKRS/TM6b, tb* (from Dr J de Navascues Melero, University of Cardiff); nab^NP3537^-Gal4 (from Prof S Russell, University of Cambridge); UAS-dGcn2-CA (from Dr P Leopold, University of Nice) [51]; *d4E-BP*^*Null*^ line (from Dr J Carmichael, University of Cambridge) [30]; *dad-GFP.N* [52]; the *uas-perk* line was described previously [14].

Unless stated otherwise, crosses were performed at 25 °C with three to four virgins and two males in standard food vials. Each 2–4 days these flies were then flipped into fresh vials to avoid overcrowding of progeny. The food used was a standard ‘lower maize, higher yeast agar’ recipe consisting of 2% (*w*/*v*) yeast, 8% (w/v) dextrose, 7% (w/v) maize and 1% (w/v) agar with the addition of nipagin and dry yeast pellets. In specific experiments modified foods were used: ’high protein food’ [5.9% (w/v) glucose, 6.6% (w/v) cornmeal, 4% (w/v) dried yeast and 0.7% agar] or ’low protein foods’ [5.9% (w/v) glucose, 6.6% (w/v) cornmeal, 0.25% (w/v) dried yeast and 0.7% agar].

For the tissue-specific screen, virgin females of the *ppp1r15* RNAi #1 or w^1118^ were crossed to males of various GAL4-driver lines. Fourteen days after egg laying (AEL), the progeny were analysed. Developmental analysis was performed as described previously [15]. To generate flip out clones in wing imaginal discs, we crossed yw.hs-flp^122^; *act5c > y*^*+*^ *> Gal4, UAS-GFP; MKRS/TM6b, tb to either w*^*1118*^ *(control), ppp1r15 RNAi* #1 or *UAS-dGcn2-CA* flies. Vials were heat shocked 4 days AEL for 15 min at 37 °C. The following day, wing imaginal discs of non-Tubby third instar larvae were dissected.

### Immunohistochemistry

Larval wing imaginal discs were dissected in phosphate-buffered saline (PBS) and fixed with 4% paraformaldehyde in PBS for 30 min at room temperature, followed by washes with PBT (PBS, 0.1% Triton X-100). For pupal wing dissections, pupae were collected at the appropriate number of hours after puparium formation (APF) and fixed with an opened case overnight at 4 °C with 4% paraformaldehyde in PBS. After dissection, an additional fixation for 30 min at room temperature was performed. Tissues were stained with the primary rabbit anti-pSMAD antibody (PS1) 1:500 (from Prof P. ten Dijke, University of Leiden) overnight at 4 °C followed by anti-rabbit Alexa 594 1:250/500 (Thermo Fisher Scientific) for 1 h at room temperature. Samples were mounted in ProLong Gold Antifade with 4’,6-diamidino-2-phenylindole (DAPI, Thermo Fisher Scientific). Images were taken using a Zeiss LSM880 microscope with a 20× and a 40× objective. Merged images of Z-stack focal planes were generated with ImageJ (NIH) showing maximum intensity.

### Generation of transgenic flies

The *UAS-HA-crcA* line was generated by amplification of the *HA-crcA* sequence from the construct HA-crcA.pMT-Puro and directionally cloned between *Not*I and *Xho*I into pUASTattB. Microinjection was performed by the Department of Genetics core facility, University of Cambridge, and stock number 13-14 yielded an insertion on the third chromosome (86F8).

### Expression plasmids

The HA-tag sequence was directionally cloned between *Bam*HI and *Eco*RI into pcDNA3.1 (HA.pcDNA3.1) and then subcloned between *Kpn*I and *Xho*I of the pMT-Puro vector (Addgene 17,923) to generate HA.pMT-Puro. The crc transcript A coding sequence was amplified from cDNA clone RH01327 (Drosophila Genomics Research Center (DGRC), Indiana University, Bloomington, IN, USA) and directionally cloned between *Eco*RI and *Xho*I into the HA.pcDNA3.1 plasmid; then HA-crcA was subcloned between *Kpn*I and *Xho*I into the pMT-Puro vector (Addgene 17,923) to generate HA-crcA.pMT-Puro. To generate dGCN2-CA-V5.pMT-Puro, the *gcn2* coding sequence was amplified from the cDNA clone AT10027 (DGRC) and mutated to incorporate an activating mutation in the translated protein (F751 L) and then cloned into the pMT-Puro vector (from David Sabatini, Addgene stock 17,923). To generate the 5’UTR-*crcE-luciferase* reporter construct, a synthesised DNA fragment (GeneArt, Thermo Fisher) containing the 5’UTR of *crcE* and the first three amino acids of the protein coding sequence was cloned in frame into a luciferase-pcDNA3.1 plasmid [15] by Gibson assembly. The crc-pGEX-6P-1 expression construct was generated by amplifying the crcA coding sequence from the cDNA clone RH01327 (DGRC) followed by cloning between *Sal*I and *Not*I in pGEX-6P-1 (Invitrogen). The construct pAFW-MAD-FLAG [53] was used to express MAD-FLAG; the construct myc-tkv.pAc5.1 was used to express myc-Tkv and was generated from the myc-tkv.pMT plasmid [54]. For punt-V5 expression, the punt coding sequence was amplified from plasmid FMO13005 (DGRC) and cloned between *Kpn*I and *Xho*I into the pAc5.1 plasmid (Thermo Fisher); for myc-sax expression, the sax coding sequence was amplified from plasmid 02439 (DGRC) and similarly cloned in pAc5.1.

### S2 cell culture

Cycloheximide was from Sigma-Aldrich; dpp was from R&D Systems. *Drosophila* Schneider 2 (S2) cells (from Dr J Hirst, Cambridge) were grown at 25 °C in Schneider medium (Sigma-Aldrich) supplemented with 10% fetal bovine serum (FBS, Invitrogen) and 100 U/ml streptomycin/penicillin (Sigma-Aldrich). Transfection reagent TransIT 2020 (Mirus Bio) was used for all experiments. To generate stable inducible lines, S2 cells were transfected with dGCN2-CA-V5.pMT-Puro or HA-crcA.pMT-Puro constructs and cultured for 2 weeks in 4 μg/ml puromycin. In parallel, control cell lines were generated with pMT-Puro or HA.pMT-Puro. Transgene expression was induced with 0.7 mM copper sulphate. For measurement of Dpp signalling, 2.5 × 10^6^ cells per well were seeded in 6-well plates, and expression was induced for 16 h (dGCN2-CA-V5) or 24 h (HA-crcA), followed by treatment with 0.5 nM or 1 nM Dpp for 1 h. When assessing protein half-lives, S2 cells were transfected in 6-well plates with 250 ng of myc-tkv.pAC5.1, myc-sax pAC5.1 or punt-V5.pAC5.1. Twenty-four hours after transfection, cells were treated with 100 μg/ml cycloheximide for up to 12 h as indicated. To assess level of pMAD-FLAG and total MAD-FLAG, S2 cells were transfected with 1 μg of MAD-FLAG.pAFW. Twenty-four hours post-transfection, cycloheximide (14 μg/ml or 100 μg/ml as indicated) was added for the indicated times with 1 nM dpp present for the final hour.

### Microarray

dGCN2-CA-V5.pMT-Puro or HA-crcA.pMT-Puro inducible cell lines were induced with 0.7 mM copper sulphate for indicated times. pMT-Puro and HA-pMT-Puro lines were induced with 0.7 mM copper sulphate for control purposes. Total RNA was prepared from cells by homogenisation and extraction using TRIzol reagent (GibcoBRL). Each total RNA sample (50 μg) was subjected to reverse transcription and direct labelling with Cy3- or Cy5-deoxycytidine triphosphates (dCTPs, Amersham). Appropriate Cy3-dCTP- or Cy5-dCTP-labelled samples were mixed together and hybridised to the International *Drosophila* Array Consortium (INDAC) oligo array FL003 for 16 h at 51 °C (Genetics core facility, University of Cambridge, UK). After hybridisation, slides were washed, spun dry and scanned with 635-nm and 532-nm lasers using a Genepix 4000B scanner (Axon Instruments). Spot intensities were normalised using variance stabilisation [55] in the Vsn package in R/Bioconductor. The magnitude and significance of each spot intensity were estimated using linear models in the LIMMA package in R/Bioconductor. False discovery rates (FDRs) were calculated using the Benjamini-Hochberg method [56]. Differentially expressed genes (exhibiting log_2_-fold changes ≤ 0.7 or > 0.7 and an FDR-adjusted *P* value of < 0.05) were subjected to GO and Kyoto Encyclopedia of Genes and Genomes (KEGG) pathway enrichment analysis using FlyMine [57].

### Immunoblotting

S2 cells were lysed in radioimmunoprecipitation assay (RIPA) buffer (50 mM Tris-HCl pH 7.4; 150 mM NaCl; 1% NP-40; 0.5% sodium deoxycholate; 0.1% sodium dodecyl sulphate (SDS); 2 mM ethylenediaminetetraacetic acid (EDTA)) supplemented with 1 mM phenylmethylsulphonyl fluoride (PMSF) and EDTA-free protease inhibitors (Sigma-Aldrich). Commercially available primary antibodies used were rabbit anti-phospho-SMAD 1/5 (which recognises *Drosophila* pMAD; 9516; Cell Signaling Technology, Danvers, MA, USA); rabbit anti-Actin (A2066; Sigma-Aldrich); rabbit 4E-BP (4923; Cell Signaling Technology).

### crc antibody preparation

BL21(DE3) pLysS *Escherichia coli* were transformed with crc-pGEX-6P-1 and then treated overnight at 37 °C with 1 mM isopropyl β-d-1-thiogalactopyranoside (IPTG) to induce expression. Recombinant protein was purified on Glutathione Sepharose 4B resin and eluted with PreScission Protease (GE Healthcare). Rabbit polyclonal antibodies were generated by Cambridge Research Biochemicals, Billingham, UK, using this antigen.

### *In situ* hybridisation

The 3’UTR of *crcA* was amplified (residues 1405–1900) from the *crcA* cDNA clone RH01327 (DGRC) and cloned into pcDNA3 (Invitrogen) by Gibson assembly (New England Biolabs, Ipswich, MA, USA). Antisense and sense digoxigenin (DIG)-labelled RNA probes were synthesised from linearised plasmid DNA using an SP6/T7 DIG-RNA labelling kit (Roche Molecular Biochemicals, Mannheim, Germany). Wing imaginal discs and pupal wings were dissected in PBS and fixed in 4% paraformaldehyde in PBS for 20 min at room temperature, and then washed twice with PBT and once with methanol. Fixed samples were washed twice with ethanol and incubated in a mixture of xylene and ethanol (1:1 *v*/v) for 60 min, washed twice in ethanol and rehydrated by immersion in a graded methanol series (80%, 50%, 25% v/v in water) and then water. Samples were treated with acetone (80%) at −20 °C and then washed twice with PBT. They were fixed again in 4% paraformaldehyde before being washed further with PBT then incubated at room temperature with 1:1 PBT:hybridisation buffer (HB, 50% formamide, 5X SSC, 5X Denhardt’s solution, 0.1% Tween 20, 100 μg/ml yeast tRNA, RNAse-free water). They were pre-hybridised for 3 h in HB at 60 °C. Sense and antisense riboprobes were diluted 1:1000 in HB and denatured at 80 °C. Samples were hybridised with diluted riboprobes at 60 °C for 18 h. The following day, samples were washed with HB solution at 60 °C and then sequentially in 50% and 25% HB solution (*v*/v) in PBT. Following further washes in PBT, the hybridised probes were detected using anti-DIG-alkaline phosphatase conjugated sheep IgG (Fab fragments) secondary antibody using nitro-blue tetrazolium (NBT)/5-bromo-4-chloro-3’-indolyphosphate (BCIP) chromogenic substrates (Roche Molecular Biochemicals).

### ^35^S labelling of cultured S2 cells

Expression of dGCN2-CA-V5 or HA-crc was induced in stable S2 cell lines by treatment with 0.7 mM copper sulphate. Thirty minutes prior to cell harvest, ten million cells were washed in PBS and resuspended in 1 ml of cysteine- and methionine-free Dulbecco’s modified Eagle’s medium (DMEM) (MP Biomedicals, Santa Ana, CA, USA; Cat.1642454) supplemented with 10% dialysed FBS and 10% Schneider medium. ^35^S-labelled cysteine and methionine Easy Tag Express Protein Labeling Mix (Perkin Elmer) were added to cells for the final 10 min of the time course before addition of 20 μg/ml cycloheximide and incubation on ice. Cells were harvested and washed in cold PBS containing 20 μg/ml cycloheximide, then lysed in harvest buffer (hydroxyethyl piperazineethanesulfonic acid (HEPES) pH 7.9, 10 mM; NaCl 50 mM; sucrose 0.5 M; EDTA 0.1 mM; 0.5% v/v Triton X-100) supplemented with protease inhibitor cocktail (Roche, Welwyn Garden City, UK) and 1 mM PMSF. Post-nuclear supernatants were separated by SDS-PAGE on 12.5% acrylamide gels and stained with InstantBlue Coomassie stain (Expedeon, San Diego, CA, USA). ^35^S incorporation was analysed by exposure to a phosphor storage plate.

### Luciferase assay

To analyse the regulatory function of the 5’UTR of the *crcE* mRNA, HEK293T cells were transfected with Luc-pcDNA3.1 or 5’UTRcrcE-Luc.pcDNA3.1 constructs and TK-Renilla luciferase plasmid as a transfection control. Six hours post-transfection, cells were treated for 16 h with tunicamycin (2.5 μg/ml) and/or ISRIB (45 ng/ml). Control cells were treated with the appropriate vehicle controls. A Dual-Glo® Luciferase Reporter Assay (Promega, Southampton, UK) was subsequently run according to the manufacturer’s instructions to quantify the fold induction of luciferase upon drug treatment. The ratio of firefly/*Renilla* luciferase luminescence was calculated and expressed as a fold change compared to that of untreated samples.

## Additional files

Additional file 1: **Figure S1.** Modulation of the ISR delays developmental delay and causes wing venation defects. (A) Phenotypes of animals expressing *ppp1r15* RNAi under the control of a panel of tissue-selective drivers. (B) Representative photomicrographs (5× objective) of *w*^*1118*^*;esgGAL4* (*esg*) and *esgGAL4 > UAS-ppp1r15* RNAi (*esg* > *ppp1r15* RNAi) animals at 5 and 14 days after egg laying (AEL). Scale bar = 1 mm. (C) Representative photomicrographs of w^1118^;*esgGAL4* (*esg*), *esgGAL4 > UAS-ppp1r15* RNAi (*esg > ppp1r15* RNAi), *esgGAL4 > UAS-gcn2* RNAi (*esg > gcn2* RNAi) and *esgGAL4 > UAS-gcn2;UAS-ppp1r15* RNAi (*esg > ppp1r15* RNAi;*gcn2* RNAi) animals at 14 days AEL. (D) Quantification of indicated crosses at days 5 and 14 AEL. *esgGAL4 > UAS-ppp1r15* RNAi (*esg > ppp1r15* RNAi), *esgGAL4 > UAS-dGCN2* RNAi (*esg > gcn2* RNAi) and *esgGAL4 > UAS-dPERK* RNAi (*esg > perk* RNAi). *n* denotes number of animals counted. *P* values calculated using *Χ*^2^ statistic with Bonferroni correction for multiple comparisons. (E) Quantification of indicated crosses at days 5 and 14 AEL. *enGAL4 > UAS-ppp1r15* RNAi (*en > ppp1r15* RNAi), *enGAL4 > UAS-gcn2* RNAi (*en > gcn2* RNAi) and *enGAL4 > UAS-perk* RNAi (*en > perk* RNAi). *n* denotes number of animals counted. *P* values calculated using *Χ* ^2^ statistics with Bonferroni correction for multiple comparisons. (F) Representative photomicrographs of adult wings of the indicated genotypes. Scale bars = 250 μm. (PDF 1057 kb)

Additional file 2: **Figure S2.** crc is *Drosophila* ATF4. (A) HA-crc-expressing S2 lysates and matched samples incubated with λ phosphatase (λ ppase) were subjected to SDS-PAGE and transferred to nitrocellulose. Immunoblotting was performed using an anti-HA antibody. (B) The 5’UTR of *crc* transcript E: small upstream open reading frames (uORFs) in *orange*; coding sequence in *red*. (C) Luminescence signal of luciferase control (*blue bars*) or 5’UTR-*crcE*-luciferase reporter (*red bars*) expressed in HEK293T cells presented as the ratio of firefly/*Renilla* luminescence fold change compared to vehicle-treated samples. Cells were treated with the indicated concentrations of ISRIB and/or tunicamycin for 16 h. Mean ± standard error of the mean (SEM). *n* = 3. *P* value calculated using ANOVA with Bonferroni *post hoc* testing. (D) Representative photomicrographs of adult eyes. *Gmr* (*gmrGAL4* driver control), *gmr > perk* (*gmrGAL4 > UAS-perk)*, *gmr > crc* RNAi (*gmrGAL4 > UAS-crc* RNAi) and *gmr > perk*;*crc RNAi* (*gmrGAL4 > UAS-crc* RNAi*;UAS-perk)*. Scale bar = 200 μm. (E) Representative photomicrographs (5× objective) of adult wings of the indicated genotypes. *en* (enGAL4 driver control), *en > dicer2*;*ppp1r15* RNAi (*enGAL4 > UAS-dicer2;UAS-ppp1r15* RNAi), *en > dicer2;crc RNAi* (*enGAL4 > UAS-dicer2;UAS-crc RNAi)* and *en > dicer2;crc RNAi;ppp1r15* RNAi (*enGAL4 > UAS-dicer2;UAS-crc RNAi;UAS-ppp1r15* RNAi*)*. *Lower panels* are enlargements of the crossvein territories. Scale bars = 250 μm. (F) Quantification of ACV phenotype in (E). (G) Representative photomicrographs of adult wings of the indicated genotypes. *nab* (*nabGAL4* driver control), *nab > ppp1r15* RNAi (*nabGAL4 > UAS-ppp1r15* RNAi), *nab > crc RNAi* (*nabGAL4 > UAS-crc* RNAi) and *nab > ppp1r15* RNAi*;crc* RNAi (*nabGAL4 > UAS-crc* RNAi*;UAS-ppp1r15* RNAi). *Lower panels* are enlargements of the crossvein territories. Scale bars = 250 μm. (H) *In situ* hybridisation of w^1118^ wing imaginal disc with sense or antisense probes to residues 1405–1900 of *crc* transcript A. (I) Representative photomicrographs of adult wings of the indicated genotypes. *nab* (*nabGAL4* driver control), *nab > crc* (*nabGAL4 > UAS-crcA)*. *Lower panels* are enlargements of the crossvein territories. Scale bar = 250 μm. (PDF 3370 kb)

Additional file 3: **Figure S3.** crc regulates genes involved in translation including 4E-BP. (A, B) KEGG pathway analysis performed on microarray data HA-crcA.pMT-Puro S2 stable cells relative to HA.pMT-Puro S2 stable cells, each treated with 0.7 mM CuSO_4_ for 3 h or 6 h to identify pathways significantly enriched within the list of differentially expressed up- or down-regulated genes with fold change of at least 1.62. Similar analysis was performed on microarrays of dGCN2-CA-V5.pMT-Puro S2 stable cells at 12 h. (C) Venn diagram to illustrate “Translation” Gene Ontology (GO) term genes induced by dGCN2, crc or both. (D) *d4E-BP* (*Thor*) mRNA level following expression of crc for the indicated times. (E) Immunoblot of cell lysates of cells expressing crc for the indicated times. (F) Effect of *d4E-BP* RNAi (16 h) on MAD phosphorylation over a range of dpp concentrations (1 h treatment). (PDF 2491 kb)

Additional file 4: **Tables S1–S15.** Analysis of transcriptional data. **Table S1.** mRNAs induced in S2 cells expressing HA-crc for 3 h. **Table S2.** mRNAs induced in S2 cells expressing HA-crc for 6 h. **Table S3.** mRNAs repressed in S2 cells expressing HA-crc for 3 h. **Table S4.** mRNAs repressed in S2 cells expressing HA-crc for 6 h. **Table S5.** Gene Ontology (GO) term enrichment of mRNAs induced in S2 cells expressing HA-crc for 3 h. **Table S6.** GO term enrichment of mRNAs induced in S2 cells expressing HA-crc for 6 h. **Table S7.** GO term enrichment of mRNAs repressed in S2 cells expressing HA-crc for 3 h. **Table S8.** GO term enrichment of mRNAs repressed in S2 cells expressing HA-crc for 6 h. **Table S9.** mRNAs induced in S2 cells expressing dGCN2-CA-V5 for 6 h. **Table S10.** mRNAs induced in S2 cells expressing dGCN2-CA-V5 for 12 h. **Table S11.** mRNAs repressed in S2 cells expressing dGCN2-CA-V5 for 12 h. **Table S12.** mRNAs repressed in S2 cells expressing dGCN2-CA-V5 for 12 h. **Table S13.** GO term enrichment of mRNAs induced in S2 cells expressing dGCN2-CA-V5 for 6 h. **Table S14.** GO term enrichment of mRNAs induced in S2 cells expressing dGCN2-CA-V5 for 12 h. **Table S15.** GO term enrichment of mRNAs repressed in S2 cells expressing dGCN2-CA-V5 for 12 h. (XLSX 284 kb)

## Abbreviations

ACV: anterior cross vein
AEL: after egg laying
ATF4: activating transcription factor 4
BMP: bone morphogenetic protein
bZIP: basic leucine zipper
CHOP: C/EBP-homologous protein
CNS: central nervous system
Crc: cryptocephal
Dpp: decapentaplegic
eIF2α: eukaryotic translation initiation factor 2 alpha
GADD34: growth arrest and DNA damage 34
GCN2: general control nonderepressible 2
GFP: green fluorescent protein
ISR: integrated stress response
MAD: mothers against decapentaplegic
PAH: pulmonary arterial hypertension
PCV: posterior cross vein
PERK: protein kinase R-like endoplasmic reticulum kinase
PPP1R15: protein phosphatase 1 regulatory subunit 15
PVOD: pulmonary veno-occlusive disease
UTR: untranslated region
4E-BP: eukaryotic translation initiation factor 4E binding protein
