# Supplementary material for: The integrated stress response regulates BMP signalling through effects on translation
Source: BMC Biol. 2018 Apr 3;16:34. doi: 10.1186/s12915-018-0503-x (PMC5881181; doi:10.1186/s12915-018-0503-x)

Supplementary Figure 1

a

Driver	Tissue	dPPP1R15 RNAi
tub-Gal4	Ubiquitous	embryonic lethal
Da-Gal4	Ubiquitous	small delayed larvae
actin-Gal4	Ubiquitous	small delayed larvae
e22c-Gal4	Ectoderm	embryonic lethal
CG-Gal4	Fat body	no phenotype
MHC-Gal4	Somatic muscle	no phenotype
Mef2-Gal4	Somatic muscle	no phenotype
fkh-Gal4	Salivary gland	no phenotype
twist-Gal4	Midgut visceral mesoderm, muscle	no phenotype
APPL-Gal4	CNS	no phenotype
179Y-Gal4	CNS	no phenotype
elav-Gal4	CNS	no phenotype
dILP2-3-Gal4	CNS, ring gland, heart	no phenotype
dILP2- Gal4	CNS, ring gland, heart	no phenotype
GMR-Gal4	Eye imaginal disc	no phenotype
esg-Gal4	Gut, larval histoblasts, CNS and imaginal discs	large delayed larvae
en-Gal4	Wing imaginal disc (posterior compartment)	delayed pupation

b

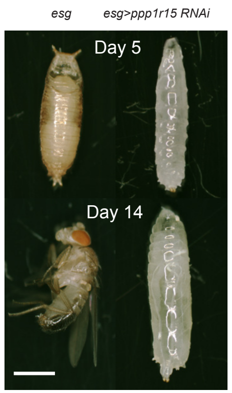

c

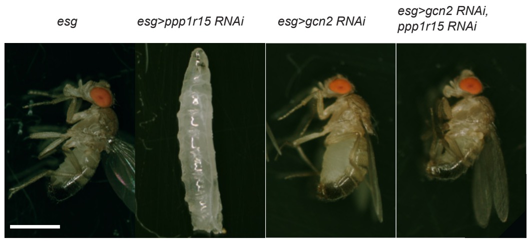

d

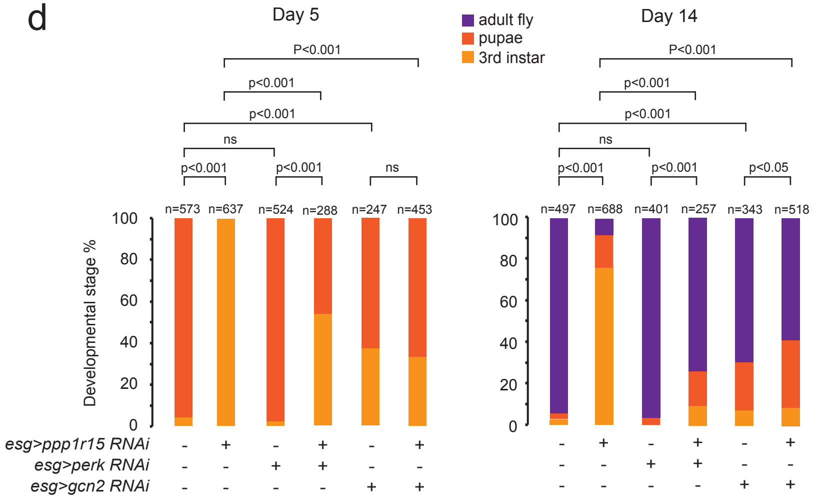

e

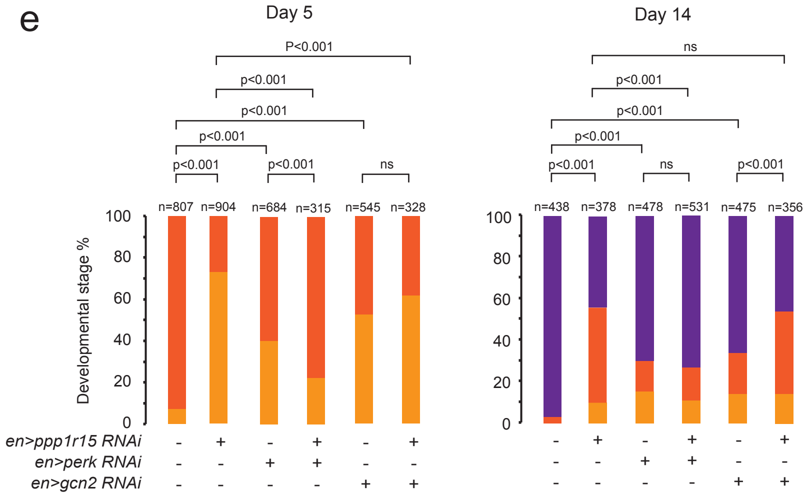

f

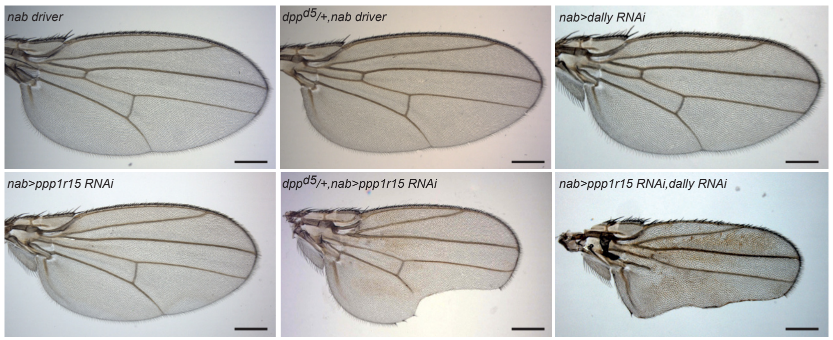

Supplement: Supplementary file 1 — Figure S1. Modulation of the ISR delays developmental delay and causes wing venation defects. (A) Phenotypes of animals expressing ppp1r15 RNAi under the control of a panel of tissue-selective drivers. (B) Representative photomicrographs (5× objective) of w1118;esgGAL4 (esg) and esgGAL4 > UAS-ppp1r15 RNAi (esg > ppp1r15 RNAi) animals at 5 and 14 days after egg laying (AEL). Scale bar = 1 mm. (C) Representative photomicrographs of w1118;esgGAL4 (esg), esgGAL4 > UAS-ppp1r15 RNAi (esg > ppp1r15 RNAi), esgGAL4 > UAS-gcn2 RNAi (esg > gcn2 RNAi) and esgGAL4 > UAS-gcn2;UAS-ppp1r15 RNAi (esg > ppp1r15 RNAi;gcn2 RNAi) animals at 14 days AEL. (D) Quantification of indicated crosses at days 5 and 14 AEL. esgGAL4 > UAS-ppp1r15 RNAi (esg > ppp1r15 RNAi), esgGAL4 > UAS-dGCN2 RNAi (esg > gcn2 RNAi) and esgGAL4 > UAS-dPERK RNAi (esg > perk RNAi). n denotes number of animals counted. P values calculated using Χ2 statistic with Bonferroni correction for multiple comparisons. (E) Quantification of indicated crosses at days 5 and 14 AEL. enGAL4 > UAS-ppp1r15 RNAi (en > ppp1r15 RNAi), enGAL4 > UAS-gcn2 RNAi (en > gcn2 RNAi) and enGAL4 > UAS-perk RNAi (en > perk RNAi). n denotes number of animals counted. P values calculated using Χ 2 statistics with Bonferroni correction for multiple comparisons. (F) Representative photomicrographs of adult wings of the indicated genotypes. Scale bars = 250 μm. (PDF 1057 kb) [file 12915_2018_503_MOESM1_ESM.pdf]
