# Supplementary figures and images for: The integrated stress response regulates BMP signalling through effects on translation
Source: BMC Biol. 2018 Apr 3;16:34. doi: 10.1186/s12915-018-0503-x (PMC5881181; doi:10.1186/s12915-018-0503-x)

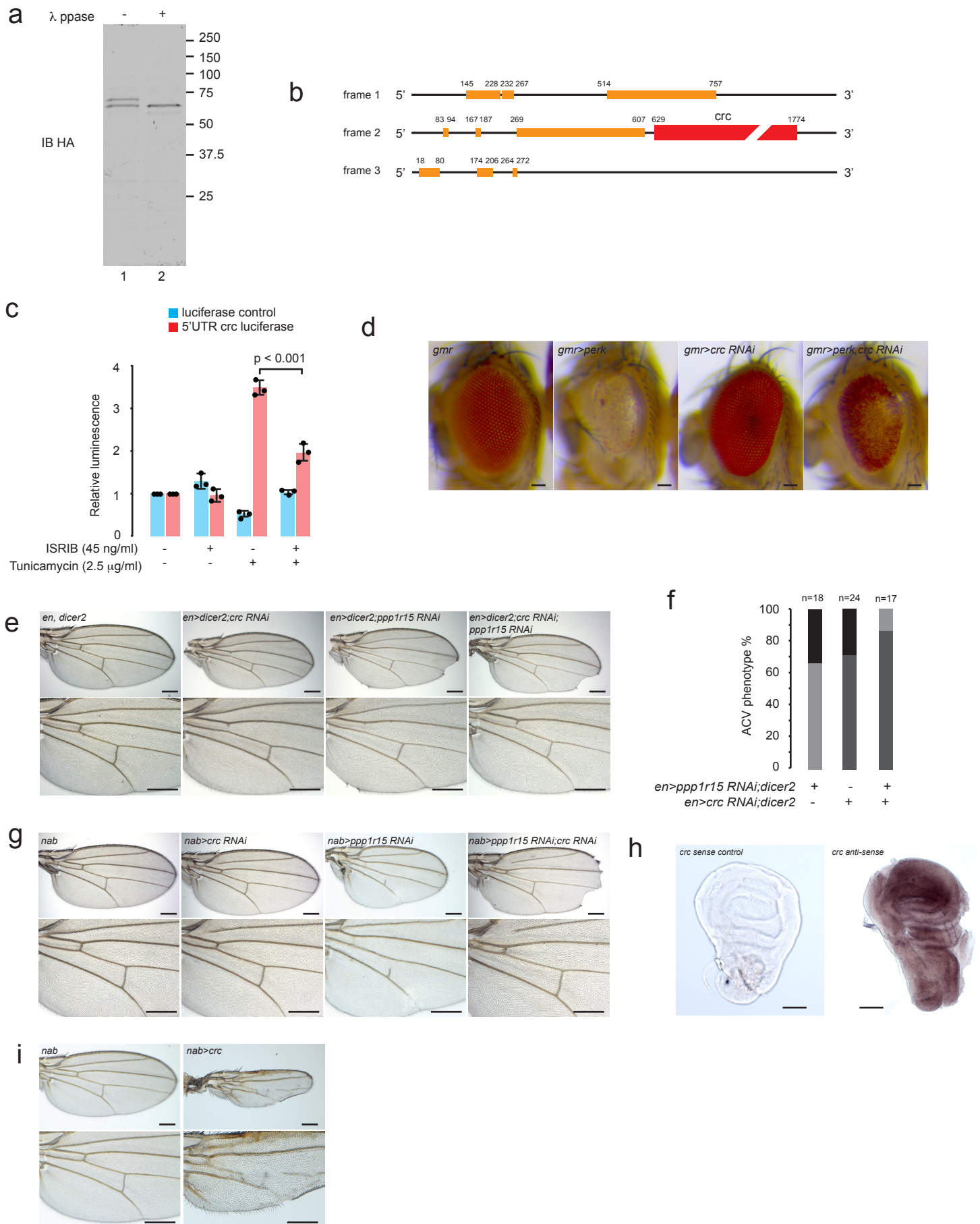

Supplement: Supplementary file 2 — Figure S2. crc is Drosophila ATF4. (A) HA-crc-expressing S2 lysates and matched samples incubated with λ phosphatase (λ ppase) were subjected to SDS-PAGE and transferred to nitrocellulose. Immunoblotting was performed using an anti-HA antibody. (B) The 5’UTR of crc transcript E: small upstream open reading frames (uORFs) in orange; coding sequence in red. (C) Luminescence signal of luciferase control (blue bars) or 5’UTR-crcE-luciferase reporter (red bars) expressed in HEK293T cells presented as the ratio of firefly/Renilla luminescence fold change compared to vehicle-treated samples. Cells were treated with the indicated concentrations of ISRIB and/or tunicamycin for 16 h. Mean ± standard error of the mean (SEM). n = 3. P value calculated using ANOVA with Bonferroni post hoc testing. (D) Representative photomicrographs of adult eyes. Gmr (gmrGAL4 driver control), gmr > perk (gmrGAL4 > UAS-perk), gmr > crc RNAi (gmrGAL4 > UAS-crc RNAi) and gmr > perk;crc RNAi (gmrGAL4 > UAS-crc RNAi;UAS-perk). Scale bar = 200 μm. (E) Representative photomicrographs (5× objective) of adult wings of the indicated genotypes. en (enGAL4 driver control), en > dicer2;ppp1r15 RNAi (enGAL4 > UAS-dicer2;UAS-ppp1r15 RNAi), en > dicer2;crc RNAi (enGAL4 > UAS-dicer2;UAS-crc RNAi) and en > dicer2;crc RNAi;ppp1r15 RNAi (enGAL4 > UAS-dicer2;UAS-crc RNAi;UAS-ppp1r15 RNAi). Lower panels are enlargements of the crossvein territories. Scale bars = 250 μm. (F) Quantification of ACV phenotype in (E). (G) Representative photomicrographs of adult wings of the indicated genotypes. nab (nabGAL4 driver control), nab > ppp1r15 RNAi (nabGAL4 > UAS-ppp1r15 RNAi), nab > crc RNAi (nabGAL4 > UAS-crc RNAi) and nab > ppp1r15 RNAi;crc RNAi (nabGAL4 > UAS-crc RNAi;UAS-ppp1r15 RNAi). Lower panels are enlargements of the crossvein territories. Scale bars = 250 μm. (H) In situ hybridisation of w1118 wing imaginal disc with sense or antisense probes to residues 1405–1900 of crc transcript A. (I) Representative photomicrog [file 12915_2018_503_MOESM2_ESM.pdf]

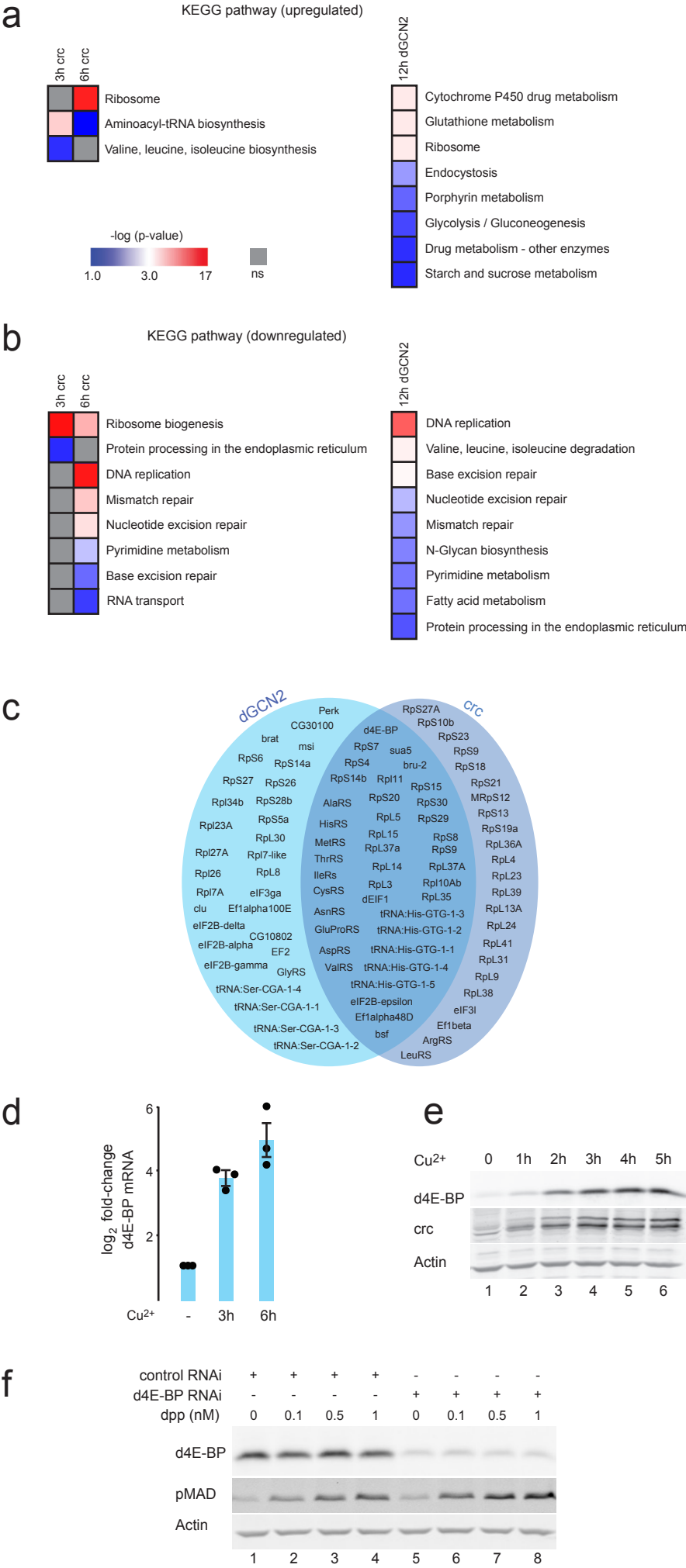

Supplement: Supplementary file 3 — Figure S3. crc regulates genes involved in translation including 4E-BP. (A, B) KEGG pathway analysis performed on microarray data HA-crcA.pMT-Puro S2 stable cells relative to HA.pMT-Puro S2 stable cells, each treated with 0.7 mM CuSO4 for 3 h or 6 h to identify pathways significantly enriched within the list of differentially expressed up- or down-regulated genes with fold change of at least 1.62. Similar analysis was performed on microarrays of dGCN2-CA-V5.pMT-Puro S2 stable cells at 12 h. (C) Venn diagram to illustrate “Translation” Gene Ontology (GO) term genes induced by dGCN2, crc or both. (D) d4E-BP (Thor) mRNA level following expression of crc for the indicated times. (E) Immunoblot of cell lysates of cells expressing crc for the indicated times. (F) Effect of d4E-BP RNAi (16 h) on MAD phosphorylation over a range of dpp concentrations (1 h treatment). (PDF 2491 kb) [file 12915_2018_503_MOESM3_ESM.pdf]
